# GCF^2^-Net: global-aware cross-modal feature fusion network for speech emotion recognition

**DOI:** 10.3389/fnins.2023.1183132

**Published:** 2023-05-04

**Authors:** Feng Li, Jiusong Luo, Lingling Wang, Wei Liu, Xiaoshuang Sang

**Affiliations:** ^1^Department of Computer Science and Technology, Anhui University of Finance and Economics, Anhui, China; ^2^School of Information Science and Technology, University of Science and Technology of China, Anhui, China

**Keywords:** speech emotion recognition, global-aware, feature fusion network, wav2vec 2.0, cross-modal

## Abstract

Emotion recognition plays an essential role in interpersonal communication. However, existing recognition systems use only features of a single modality for emotion recognition, ignoring the interaction of information from the different modalities. Therefore, in our study, we propose a global-aware Cross-modal feature Fusion Network (GCF^2^-Net) for recognizing emotion. We construct a residual cross-modal fusion attention module (ResCMFA) to fuse information from multiple modalities and design a global-aware module to capture global details. More specifically, we first use transfer learning to extract wav2vec 2.0 features and text features fused by the ResCMFA module. Then, cross-modal fusion features are fed into the global-aware module to capture the most essential emotional information globally. Finally, the experiment results have shown that our proposed method has significant advantages than state-of-the-art methods on the IEMOCAP and MELD datasets, respectively.

## 1. Introduction

In recent years, speech plays an important role in daily communication. It contains not only the textual content but also the emotional message that the speaker intends to convey (Sreeshakthy and Preethi, 2016). The same text with different tones of voice conveys different emotions. To improve the convenience of life, speech emotion is widely used in the field of HCI (Hartmann et al., 2013). Take the ubiquitous virtual voice assistants (such as Alexa, Siri, Google Assistant and Cortana), they must infer the user's emotions and respond appropriately to enhance the user experience (Dissanayake et al., 2022). In the driver emotion detection system, when the system detects that the driver is excited, exhilarated, depressed or tired, a safety alert will be issued in time to avoid traffic accidents. The online distance-assisted teaching system allows teachers to identify the emotional state of students in order to adjust the teaching style and pace. In addition, emotion recognition has important implications in healthcare. It can help doctors better understand the patient's psychological state to facilitate recovery. However, most HCI products obey the external commands of humans in a foolish way. There are few HCI products that can determine the inner emotional state of humans from external commands. Therefore, how to make machines correctly recognize emotional states needs further research, and speech emotion recognition needs more attention (Schuller, 2018; Fan et al., 2022).

However, humans express emotions not only through speech but also in many other ways, such as text, body gestures, facial expressions (Zhang et al., 2022), and electroencephalography (EEG) (Chang et al., 2022, 2023; Han et al., 2022). Chakravarthi et al. (2022) proposed an automated CNN-LSTM with the ResNet-152 algorithm to identify emotional states from EEG signals. Additionally, Wu et al. (2023) developed a novel experimental paradigm that allows odors dynamically participate in different stages of video-evoked emotions, to investigate the efficiency of olfactory-enhanced videos in inducing subjects' emotions. Thus, understanding the emotions expressed in an utterance requires a comprehensive understanding of various modalities. People also usually use changes in body movements to express emotions, for example, when the head sinks between the shoulders, no movement or crouching will be recognized as fear. Facial emotion recognition has become an important topic in the field of computer vision and artificial intelligence due to its great academic and commercial potential (Ko, 2018). Khaireddin and Chen (2021) used a single network VGGNet to achieve optimal results on the FER2013 dataset without using additional training data. Cho et al. (2019) used LSTM to extract acoustic features and parallel convolution with several different kernels to extract different levels of contextual information from word sequences. Zhao et al. (2022) proposed a multi-granularity framework that can extract frame-level speech embeddings and segment-level embeddings including phoneme, syllable, and word-level speech embeddings. Although in some cases, facial expressions can be more effective in conveying feelings. Due to difficulties in data collection, publicly available datasets often do not have enough speakers to properly cover individual differences in emotional expression. First, compared to other modalities, speech and text data are easy accessible than other data. They are also the most intuitive expressions in communication. Second, audio features and text features have extremely high similarity in time series and can be transformed into each other. Finally, audio and text are the most common modality combinations, and many advanced comparison methods exist. Therefore, in our study, we select only speech and text modalities for emotion recognition.

Speech emotion recognition uses audio signals to simulate human perception and infer emotion categories (Babu et al., 2021). Since the emotional features of various emotional speech are distinct, the machine classifies the emotions based on this variability. Speech emotion recognition is a technique that uses machines to learn the difference between emotion features and implement emotion classification by building emotion classification models. So far, speech is one of the most studied modalities in ER (Venkateswarlu et al., 2022). In the early stage, researchers proposed machine models focused on Support Vector Machines (SVM) (Jain et al., 2020), Gaussian mixture models (GMM) (Kandali et al., 2008), and Hidden Markov models (HMM) (Nwe et al., 2003). Machine models were developed using engineering features (Ververidis and Kotropoulos, 2006; Kishore and Satish, 2013), including Mel Frequency Cepstrum Coefficient (MFCC), energy, pitch, etc. Jain et al. (2020) trained SVM for emotion classification using features such as MFCC, Linear Predictive Cepstral Coefficient (LPCC), energy, pitch and speaker rate. Nwe et al. (2003) used LPCC to represent speech signals and discrete hidden Markov models as classifiers. Yang et al. (2022) utilized the self-paced regularization to find a better factorized matrices by sequentially selecting data in the learning process. Kwon et al. (2003) used quadratic discriminant analysis (QDA) and SVM to classify the extracted engineering features and demonstrated that pitch and energy are the most important factors for speech emotion recognition. The performance of traditional models is good or bad depending on the diversity of features. Meanwhile, traditional machine learning methods had proven to perform relatively well in emotion classification. Researchers continue to explore other features or algorithms to fit expressions of various emotions. With the advance in deep learning techniques, deep neural networks have achieved tremendous success (Zhang et al., 2021). Chan et al. (2016) proposed a method to transcribe speech expressions into characters for recognition. It generates character sequences without making any independence assumptions. Ramet et al. (2018) added a long and short-term memory neural network to the attention mechanism that considers temporal information in speech during the computation of the attention vector. Early engineering features have been developed with the assistance of deep neural network learning into advanced features extracted from raw waveforms (Gao et al., 2019). Gao et al. (2019) tracked continuous mood changes in arousal-valence two-dimensional space by combining raw waveform signals and spectrogram inputs. Han et al. (2014) first introduced deep learning to SER by using deep neural networks (DNNs) to extract high-level features from raw audio. It uses deep neural networks to generate probability distributions of emotional states and construct discourse-level features, which are then fed into an Extreme Learning Machine (ELM) to identify discourse-level emotions. Zhu and Li (2022) used a global-aware fusion module to capture the most important emotional information across various scales. On the other hand, transfer learning is extensively implemented in the SER field. Many approaches use pre-trained self-supervised learning functions to handle various downstream speech processing tasks such as telephone recognition (PER), automatic speech recognition (ASR), etc. Yang et al. (2021) addressed the Speech processing Universal PERformance Benchmark (SUPERB) task by learning a task-specific lightweight prediction head on top of a frozen shared model. It has recently been shown that superior speech representation can be obtained using a pre-trained wav2vec 2.0 model with learnable weighting to combine the local and contextual outputs of the models. Wang et al. (2021) explored partial tuning and overall fine-tuning of wav2vec 2.0 and HuBERT pre-trained models using a simple proposed downstream framework in three non-ASR speech tasks such as speech emotion recognition, speaker verification, and spoken language understanding. Experimental results demonstrate the superiority of the fine-tuned model in learning rhyme, vocal pattern and semantics. Chen et al. (2022) used ECAPA-TDNN as a downstream model to explore the limitations of speech representation learned through different self-supervised targets and datasets and used as an automatic speaker verification (ASV).

In some natural environments, speech is not the best choice for emotion recognition because it does not provide compelling features. Many experiments have also demonstrated that neutral recognition rates are extremely unsatisfactory compared to other emotions. In this case, text information would be a preferable choice instead of audio information for ER. Text emotion recognition (TER) aims to utilize textual information in discourse for emotion recognition. Early approaches typically used bag-of-words to present textual information and then utilized machine learning methods such as SVM and HMM to classify emotions (Zhang and Zheng, 2016). With the rise of deep learning, deep neural networks such as convolutional neural networks (CNN) and long short-term memory (LSTM) are extensively used in TER (Irsoy and Cardie, 2014; Chen et al., 2018).

Humans often use multiple methods to express their emotions simultaneously, which makes emotion recognition an inherently complex multimodal task. Speech emotion recognition requires a thorough understanding of both the linguistic content of the discourse (textual information) and the way of the speaker's pronunciation (audio information). It is an important challenge for SER to effectively fuse these two types of information (Xu et al., 2019). Audio information and text information come from different modalities. Although there exists some correlation among them, it is not an easy task to fuse them. Therefore, integrating data from other modalities is the primary problem (Cambria et al., 2017). The rise of attention mechanisms have prompted many researchers to attempt fusing information from other modalities using different attention mechanisms, such as self-attention and cross-modal attention (Cao et al., 2021; Sun et al., 2021; Wu et al., 2021). Self-attention mechanisms and cross-modal attention mechanisms target different objects. Self-attention mechanisms are typically used for unimodal emotion recognition, whereas cross-modal attention mechanisms are used for multimodal emotion recognition. Sun et al. (2021) used cross-attention and self-attention mechanisms in parallel for inter- and intra-modal interaction of audio and text. The invocation of attention mechanisms dramatically improves the performance of cross-modal models for ER. Therefore, we use the cross-modal attention (CMA) mechanism to fuse audio and text features in our study. In order to obtain more effective cross-modal fusion features, we propose a ResCMFA module based on the CMA for SER.

Although these approaches have achieved considerable success, several key issues remain to be addressed. First, the emotion of the utterance is usually closely related to the context. However, most discourse-level feature modeling approaches do not capture enough contextual information. Second, the interaction between audio and text often changes the expressed emotional state. Relying on audio or textual information alone does not provide sufficient robustness in emotion recognition. Therefore, increasing attention devote to the use of cross-modal methods. Our study compensates for the shortcomings of using only audio modalities by adding textual information to the audio information. Through cross-modal attention mechanisms, we effectively combine audio information with textual information. The inclusion of the global-aware block allows our proposed model to extract sufficient contextual information about the utterances.

In a word, in this paper, we propose a global-aware Cross-modal feature Fusion network (GCF^2^-Net) that extracts features from different modalities by constructing a residual cross-modal fusion attention (ResCMFA) layer and designing a global-aware fusion module. The ResCMFA can dynamically fuse audio and text information and the global-aware block can capture the emotional information from the fusion features at multiple scales. First, we use different pre-trained models as encoders for different modalities. In this case, the audio encoder is the wav2vec 2.0 model and the text encoder is the Roberta-base structure. Second, we designed the residual cross-modal feature fusion module (ResCMFA) based on CMA to fuse wav2vec 2.0 features and text features. It uses text features and audio features as the query, key and value of CMA, respectively. This approach maps different modal information to each other's feature potential space for generating emotionally relevant representations. The residual structure reduces the missing information caused by audio features and text features passing through crossmodal attention mechanisms, linear layers, etc. Third, we introduce a global-aware fusion module to handle the significant emotional information of different modalities. Finally, we use ASR as an auxiliary task to eliminate text contextual bias. This approach better takes into account the natural monotonic alignment between audio features and text features. Our GCF^2^-Net model achieves state-of-the-art (SOTA) results on the IEMOCAP and MELD datasets. The main contributions of the work are outlined below as a summary.

- We propose a novel cross-modal fusion network called GCF^2^-Net for recognizing different emotions in utterances. A residual cross-modal fusion attention module (ResCMFA) is constructed to fuse information from different modalities and aggregate features at different levels through residual connections.
- We design a new global-aware fusion module to grab the most crucial emotional information across multiple scales and capture global information. Our study displays the great potential of information fusion among different granularities.
- Experiments demonstrate our GCF-Net model achieves state-of-the-art ER results on the IEMOCAP and MELD datasets. On the IEMOACAP dataset, our proposed GCF-Net model improves by 1.65% in WA and 1.10% in UA compared to the state-of-the-art results. On the MELD dataset, our proposed GCF-Net model improves the accuracy by 1.90% and the weighted average F1 by 1.10% compared to the state-of-the-art results.

The rest of the paper is organized as follows: Section 2 reviews related work on unimodal and multimodal emotion recognition. Section 3 describes our proposed model in detail. Section 4 verifies the validity of each block in our model through ablation experiments, and we summarize the paper in Section 5.

## 2. Related work

### 2.1. Unimodal emotion recognition

From traditional machine learning to deep learning, speech has been the most studied modality in ER. The proposal of transfer learning has further contributed to the development of SER. Transfer learning is commonly used to solve the problem of insufficient data. Compared to other modal recognition tasks, sparse speech data can easily lead to overfitting problems as neural network models cannot learn the actual data distribution. However, the proposed wav2vec 2.0 solves this issue. The wav2vec 2.0 is a framework for obtaining speech representations through self-supervision (Baevski et al., 2020). The wav2vec 2.0 is initially applied to ASR by training on large amounts of unlabeled speech data and then fine-tuning the labeled data to achieve automatic recognize human's speech. And the wav2vec 2.0 features are rich in rhythmic information. Pepino et al. (2021) used the wav2vec 2.0 representation as the feature extractor for SER. Makiuchi et al. (2021) proposed a new cross-representation model to reconstruct low-level Mel spectrograms from wav2vec 2.0 speech representations and then combine two audio features and text features for ER. Therefore, we choose the wav2vec 2.0 model as the audio encoder. Inspired by Cai et al. (2021), we find that the selection of a suitable auxiliary method contributes to the performance of SER. In the training phase, combining the ASR task calculates the CTC loss of the minimized network to optimize the loss function.

Pang and Lee (2004) proposed a TER method that that emotion analysis aims to identify the viewpoint behind the text span and propose to apply text classification techniques to the subjective part of the document. The emergence of the transformer technology has accelerated the process of TER. There are two mainstream models for TER, including scratch-trained ASR models and word embedding models. The word embedding models include Word2Vec (Mikolov et al., 2013) and GloVe (Pennington et al., 2014). Mikolov et al. (2013) computed continuous vector representations of words, providing state-of-the-art performance to measure syntactic and semantic word similarity. Pennington et al. (2014) trained only the non-zero elements of the word-word co-occurrence matrix, rather than the entire sparse matrix or a single contextual window in a large corpus. This method effectively exploits statistical information, which produces a vector space with meaningful substructure. These word embedding models are unsupervised. They have achieved great success in emotion analysis in natural language processing (NLP). However, these models have a very significant limitation. Since these models do not consider word ordering when modeling, they lose the syntactic and semantic understanding of the words. The introduction of the bert model solves the problem (Devlin et al., 2018). Compared to word embedding models like GloVe, the transformer-based bi-directional pre-trained bert model has superior performance in terms of word representation. The bert model successfully extracts contextual representations from text data by masking the language model (MLM) pre-training target. Some researchers have even combined bert-based embedding with speech-based representation to improve the performance of ER (Pepino et al., 2020). Wu et al. (2021) proposed a novel two-branch neural network model structure consisting of temporally synchronous branching (TSB) and temporally asynchronous branching (TAB). TSB combines speech and text modalities at the input window frame and then uses pooling across time to form a single embedding vector. TAB integrates sentence text embeddings from multiple contextual discourses into another embedding vector to provide cross-talk information. Li et al. (2020) proposed a transformer-based EDC context and speaker-sensitive model by adding a transformer on the conversational side. The approach encodes individual sentences with bert and then performs multi-task learning on auxiliary tasks using dialogue-level networks to produce a better potential representation of the whole dialogue. The roberta model is an enhanced version of the bert model (Liu et al., 2019). Compared to bert, roberta's model has a larger number of model parameters and batch sizes, as well as uses more data to train. It presents a set of important bert design choices and training strategies and introduces alternatives that can improve the performance of downstream tasks. Therefore, in this paper, we choose roberta-base pre-trained model as a contextual encoder of textual information to improve the model performance for SER.

Additionally, adding extra information to the text information can also improve the performance of TER. Sheng et al. (2020) used graph neural networks to encode inter-discourse and inter-speaker relationship information and incorporated it into textual information for ER. Wang et al. (2020) used an LSTM-based encoder to encode the interlocutor itself and the interlocutor's related information. Then, multi-layer transformers are used to enhance the encoding capability of the LSTM.

### 2.2. Cross-modal emotion recognition

Cross-modal emotion recognition aims to capture sufficient emotional information from different modalities for ER. Various modalities exist in current ER research, yet the most common combination is speech and text (Girish et al., 2022). Cho et al. (2019) used LSTM to extract acoustic features and parallel convolution with several different kernels to extract different levels of contextual information from word sequences. Zhao et al. (2022) proposed a multi-granularity framework that can extract frame-level speech embeddings and segment-level embeddings including phoneme, syllable, and word-level speech embeddings. Cross-modal models are divided into early fusion and late fusion according to the time or stage of feature fusion (Tripathi et al., 2018; Wang et al., 2019). Late fusion is usually combined with the final decision scores of various modalities for emotion recognition. It is a static fusion strategy that does not generate a novel cross-modal fusion feature. Sebastian et al. (2019) used a late fusion strategy for cross-modal emotion recognition. The method uses LSTM-RNN and pre-trained word embeddings for text emotion recognition and CNN with discourse-level descriptors for speech emotion recognition. In contrast, early fusion focuses on exploring the interactions between the original features of different modalities (Poria et al., 2017). Ahn et al. (2022) proposed to circulate multi-head attention in a fusion architecture, which can select significant fusion representations and learn the dynamics.

Early fusion usually takes a tandem or attention mechanism to fuse the characteristics of different modalities. The two fusion methods have their own merits. Late fusion is effective for specific modes, but it is not entirely practical for cross-modal interactions (Georgiou et al., 2019). Previously, it was worth pointing out that early fusion was not superior to late fusion (Poria et al., 2018b) for ER. The emergence of cross-modal attention mechanisms has accelerated the development of early fusion.

Early cross-modal fusion networks only cascaded features from different modalities. This method does not effectively carry out inter-modal interactions to obtain fusion features. The cross-modal attention mechanism (CMA) can effectively infer the potential relationships among different modalities (Choi et al., 2018; Krishna and Patil, 2020). The CMA can dynamically fuse information from different modalities by simulating the interaction between the different modalities. The dynamic interaction among modalities can improve the performance of the emotion recognition system. Srivastava et al. (2022) used CMA to fuse audio and text features. Then, CTC loss was calculated using ASR as an auxiliary task for ER. Chudasama et al. (2022) used a multi-head attention mechanism to fuse three modal features: visual, audio, and text, respectively. Yoon et al. (2019) proposed a multi-hop attention mechanism to infer the inter-modal correlations through training automatically.

Therefore, to improve the performance of the proposed method, we use the CMA attention mechanism as a fuser of the different modal features in our model. The stacked combination of multiple ResCMFA blocks forms our cross-modal attention module. It provides a unique cross-modal interaction framework for speech and text modality.

## 3. Proposed methodology

**Figure 1:**
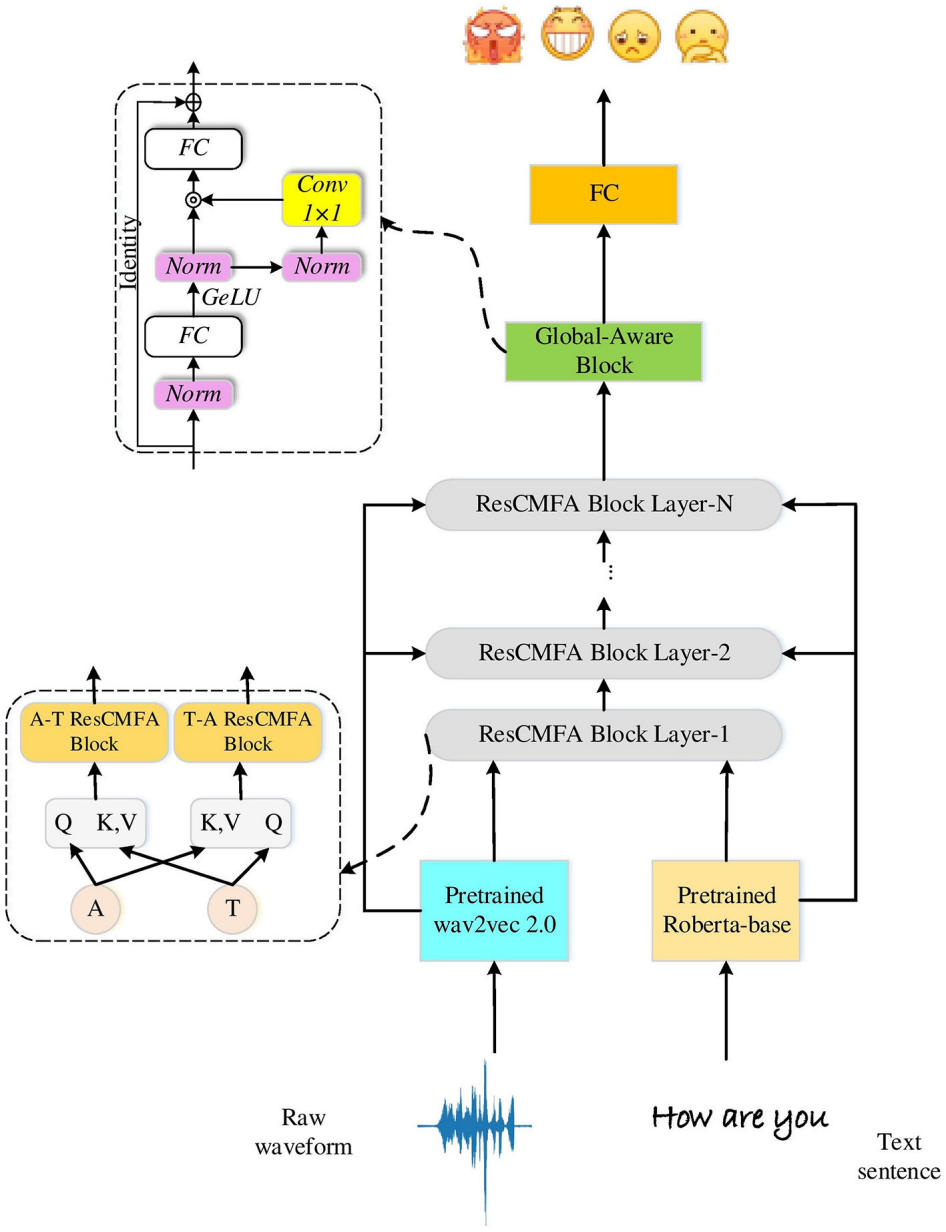
The framework of the proposed method.

This section briefly describes the architectural design details of the proposed framework. We propose a cross-modal emotion recognition model that takes speech and text from conversations as input and outputs the predicted emotions. Figure 1 shows the specific architecture of the model. It has two main parts: SER (gray) and ASR (green). SER mainly comprises two pre-trained feature extraction models (wav2vec 2.0 model for audio features and roberta-base model for text features), the ResCMFA module, and the Global-Aware block. This part calculates the CrossEntropy loss by predicting the emotion label and the true emotion label. The ASR part calculates the CTC loss through the audio features of the wav2vec 2.0 model and the corresponding text transcriptions. Finally, we feed the extracted features into the fully connected layer for classification to obtain the predicted emotion category.

### 3.1. Problem statement

The dataset D has *k* utterances **u_*i*_**, which correspond to the labels *l*_*i*_. Each utterance consists of a speech segment *a*_*i*_ and a text transcript *t*_*i*_, where **u_*i*_**∈(**a_*i*_**, **t_*i*_**). *t*_*i*_ is either an ASR transcript or a human-annotated. The proposed network takes *u*_*i*_ as input and assigns the correct emotion to any given discourse.


(1) $$\langle \text{U},\text{L}\rangle =\left\{\langle {\text{a}}_{i},{\text{t}}_{i}\rangle ,l_{i}\right\};i\in [1,k]$$

### 3.2. Feature encoder

For the audio pattern, we use a pre-trained wav2vec 2.0 model as the original audio extractor. The wav2vec2.0 model is obtained from the pre-training checkpoints released by Facebook. The wav2vec 2.0 features are rich in the rhythmic information needed for emotion recognition. Comparing the two versions of the wav2vec 2.0 model, we chose to use the wav2vec2-base architecture with a dimension size of 768. The wav2vec2-base model consists of a convolutional feature encoder and 12 stacked transformers encoders. Here, we only use the state of the last layer of the pre-trained model as input. The input audio data **a_*i*_** of the *i*^th^ utterance into the pre-trained wav2vec 2.0 model to get the contextual embedding representation $e_{i}{}^{a}$, $e_{i}{}^{a}\in {\mathbb{R}}^{j\cdot D_{W}}$. The *D*_*W*_ indicates the size of the audio feature embedding, *D*_*W*_ is 768. Thus, the $e_{i}{}^{a}$ can be expressed as follows


(2) $$\begin{array}{l} {\text{e}}_{i}^{a}=\Phi_{\text{wav2vec}2.0}\left({\text{a}}_{i}\right)\in {\mathbb{R}}^{j\times D_{W}};i\in \left[1,k\right] \end{array}$$

where Φ_wav2vec2.0_ denotes the function of the pre-trained wav2vec 2.0 model as an audio feature processor. *j* depends on the size of the raw audio and the CNN feature extraction layer in the wav2vec 2.0 model. This CNN layer extracts frames from the raw audio with a stride of 20*ms* and a hop size of 25*ms*. In our experiments, it is worth noting that the parameters of the CNN feature extraction layer are fixed at a constant level.

For the text pattern, the popular roberta is used. It contains a tokenizer and 12 transformers encoder. Compared with the common bert model, roberta uses a dynamic adjustment mask method to extract more effective text features. In our study, we also choose roberta-base as our feature extractor with a dimension size of 768. First, we should tokenize the input text data *t*_*i*_ and add separators 〈*S*〉 and 〈/*S*〉 to separate the sentences. Then, we are fine-tuning the tokenized text data and the corresponding discourse to prevent semantic confusion. Finally we feed the processed text data into the transformers encoders to generate a feature map of size m*D_*T*_. The extracted contextual embeddingA can be expressed as:


(3) $$\begin{array}{l} {\text{e}}_{i}^{t}=\Phi_{\text{Roberta-base}}\left({\text{t}}_{i}\right)\in {\mathbb{R}}^{m\times D_{T}};i\in \left[1,k\right] \end{array}$$

where Φ_Roberta-base_ denotes the text feature extraction module and *m* depends on the number of tokens in the text. And *D*_*T*_ is the dimension of text feature embedding.

### 3.3. Feature fusion

Our residual cross-modal fusion attention module consists of multiple ResCMFA block layers stacked as shown in Figure 1. The ResCMFA block layer consists of two parallel fusion blocks targeting different modalities, labeled as A-T ResCMFA block and T-A ResCMFA block. The difference between the two ResCMFA blocks are the Query, Key, and Value of the cross-modal attention mechanism. The A-T ResCMFA block sends audio features ($e_{i}{}^{a}$) as Query, and text feature ($e_{i}{}^{t}$) as Key and Value to the cross-modal attention mechanism for the interaction of audio and text information. And the T-A ResCMFA block uses text features ($e_{i}{}^{t}$) as Query, and audio features ($e_{i}{}^{a}$) as Key, Value.

**Figure 2:**
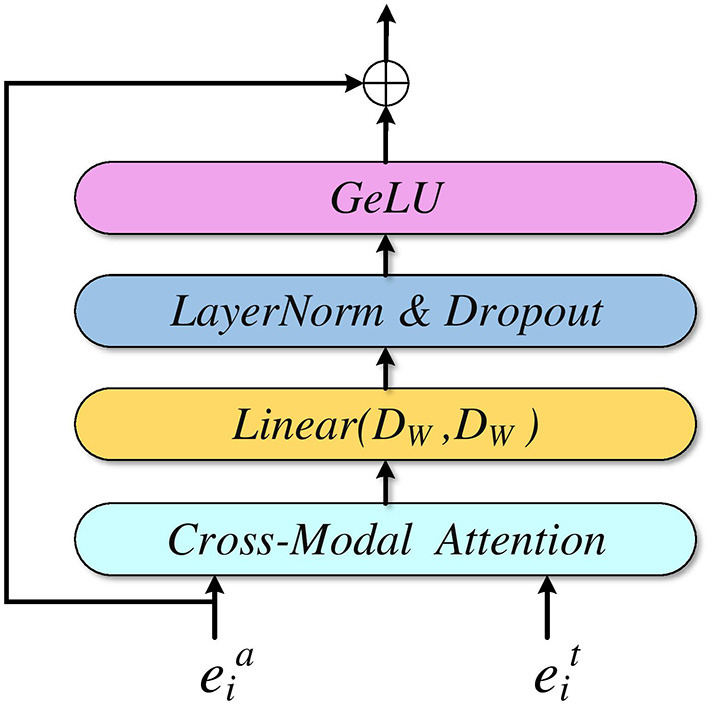
A-T residual cross-modal fusion attention block.

In Figure 2, we can see that a ResCMFA block consists of a cross-modal attention mechanism, a linear layer, layer normalization, a dropout layer and a residual structure. First, audio features and text features interact through a cross-modal attention mechanism. The CMA is based on a multi-head attention mechanism to better fuse audio features and text features. Then, the interacted features pass through a linear layer, layer normalization, GeLU and dropout layer. Finally, it is connected to the initial audio features (A-T ResCMFA block) or text features (T-A ResCMFA block) of the block through a residual structure. The residual structure can reduce the missing audio information and text information in the fusion features and protect the integrity of the information.


(4) $$\begin{array}{l} {\text{F}}_{\text{fusion}1}^{i}=\Phi_{1}\left({\text{e}}_{i}^{a},{\text{e}}_{i}^{t}\right) \end{array}$$

where Φ_1_ represents the learning function of the proposed 1st A-T ResCMFA or T-A ResCMFA.

In addition, the output of the first ResCMFA block combined with the initial audio features or text features are feed into the second ResCMFA block. In this way, multiple ResCMFA blocks are stacked together to generate the corresponding cross-modal fusion feature $F_{A-T_{m}}^{i}$ and $F_{T-A_{m}}^{i}$.


(5) $$\begin{array}{l} A-T:{\text{F}}_{A-T_{m}}^{i}=\Phi_{m}\left(\ldots \left(\Phi_{2}\left({\text{F}}_{\text{fusion}1}^{i},{\text{e}}_{i}^{t}\right)\right)\right)\text{, where}\;m\in \left[1,N\right] \end{array}$$

(6) $$\begin{array}{l} T-A:{\text{F}}_{T-A_{m}}^{i}=\Phi_{m}\left(\ldots \left(\Phi_{2}\left({\text{F}}_{\text{fusion}1}^{i},{\text{e}}_{i}^{a}\right)\right)\right),\text{where}\;m\in \left[1,N\right] \end{array}$$

Our fusion strategy is quite different from the previously proposed cross-modal fusion. To better integrate cross-modal information, we will always keep the Key and Value, the different values of each ResCMFA block as the initial audio and text feature of the module. The outputs of the last two ResCMFA blocks are concatenated to generate the final cross-modal fusion feature.


(7) $$\begin{array}{l} {\text{F}}_{\text{fusion}}^{\text{cross-modal}}=\mathrm{Concate}\left({\text{F}}_{A-T_{m}}^{i},{\text{F}}_{T-A_{m}}^{i}\right) \end{array}$$

As the ResCMFA module extracts local fusion features, we consider associative features and enhance feature communication. From Figure 1 we can see that the fused features that pass through the ResCMFA module are fed to the global-aware block (Liu et al., 2021). The global-aware block can globally capture the rich emotional information contained in the fused features, which consists of two fully connected layers, a convolutional layer, two normalization layers, a GeLU activation function and a multiplication operation. The output dimensions of the first and last fully connected layers in the module are 4*D*_*f*_ and *D*_*f*_ (the size of *D*_*f*_ is 768), respectively. After the GeLU activation function projection, the output is split into 2*D*_*f*_ along the feature size. The multiplication operation enhances feature mixing across dimensions. Finally, the output of the global-aware module is integrated for classification.


(8) $$\begin{array}{l} {\text{F}}_{\text{global-aware}}={\text{Φ}}_{\text{global-aware}}\left({\text{F}}_{\text{fusion}}^{\text{cross-modal}}\right) \end{array}$$

(9) $$\begin{array}{l} y_{i}=\text{FC}\left({\text{F}}_{\text{global-aware}}\right)\in {\mathbb{R}}^{C} \end{array}$$

where Φ_*global*−*aware*_ is the function of cross-modal fusion features through the Global-Aware block. *C* is the number of emotional categories.

### 3.4. CTC layer

In the training phase, we update the gradients by two loss functions (CrossEntropy loss and CTC loss). CTC is the standard technique for mapping input signals to output targets when they don't have the same length and no alignment information is provided. Here, the length *m* of the speech signal is usually significantly longer than the length *j* of the text transcription. CTC loss is used as a loss function to effectively back-propagate the gradient. Thus, we calculate CTC loss by waw2vec 2.0 features $e_{i}{}^{a}$ and text transcription information **t**_*i*_.


(10) $$y_{i}{}^{a}=\mathrm{sof}\mathrm{tmax}\left(e_{i}{}^{a}\right)$$

(11) $$L_{CTC}=CTC\left(y_{i}{}^{a},t_{i}\right)$$

where $y_{i}^{a}\in {\mathbb{R}}^{j*V},V=32$ is the size of our vocabulary list, consisting of 26 letters in the alphabet and a few punctuation marks. In addition, we compute the CrossEntropy loss using the output features *y*_*i*_ of the global-aware block and the true emotion label *l*_*i*_.


(12) $$\begin{array}{l} L_{\text{CrossEntropy}}=\mathrm{CrossEntropy}\left(y_{i},l_{i}\right) \end{array}$$

Finally, we introduce a hyper-parameter α that combines the two loss functions into a single loss. α can effectively control the relative importance of CTC loss.


(13) $$\begin{array}{l} L=L_{\text{CrossEntropy}}+\alpha L_{CTC},\alpha \in \left(0,1\right) \end{array}$$

## 4. Experimental evaluation

### 4.1. Experiment dataset

Under many existing techniques in SER literature, we trained and evaluated all our models on the IEMOCAP and MELD (Poria et al., 2018a) datasets. The IEMOCAP dataset, a multimodal dataset, is the benchmark for emotion recognition research. It contains 12 h of improvised and scripted audio-visual data from 10 UC theater actors (five males and five females) in five binary sessions. The emotional information of each conversation is presented in four ways: video, audio, transcription, and motion capture of facial movements. In this experiment, we select audio and transcript data to evaluate our model on the IEMOCAP dataset. Like the majority of studies, we choose five emotions for ER: happy, angry, neutral, sad, and excited. Since happy and excited are highly similar, we label all excited sample data as happy. A total of 5531 data are available and Table 1 shows the statistics of the IEMOCAP dataset. We randomly divide the dataset into training (80%) and testing (20%) parts with five-fold cross-validation to evaluate our model (Xu et al., 2021).

**Table 1:** Statistics of the IEMOCAP dataset.

**Emotion**	**Number**
Anger	1,103
Neutral	1,708
Happy	595
Excited	1,041
Sad	1,084

The MELD dataset is derived from more than 1,400 dialogues and 13,000 utterances from the TV-series Friends. Each utterance is annotated by one of seven emotion labels (e.g., neutral, surprise, fear, sadness, joy, disgust, anger). This dataset is extended for multimodal scenes and it is the most used benchmark dataset for multimodal emotion recognition. We split the dataset using the predefined training/validation provided on the MELD dataset. The details of the MELD dataset are shown in Table 2.

**Table 2:** Statistics of the MELD dataset.

**Emotion**	**Train + ev**	**Test**
Neutral	5,180	1,256
Surprise	1,355	281
Fear	308	50
Sadness	795	208
Joy	1,906	402
Disgust	293	63
Anger	1,261	345

### 4.2. Experiment setup

In this work, to explore the advantages of multimodality, we construct two unimodal baselines using text and speech modalities. Text baseline using roberta-base as the contextualized text encoder, then classification using a single linear layer and softmax activation function. The speech baseline used a similar setup as the text baseline, replacing only the encoder with a pre-trained wav2vec 2.0 model. We use the Pytorch framework to build our model.

To compare with other approaches, we set the same hyperparameters settings in this experiment. First, the batch size is set to 2. In the training part, we calculate the loss by CrossEntropy loss and CTC loss, and update the parameters of the model by Adam optimizer with learning rate of 1e-5. The α is used to control the intensity of CTC loss. For the testing part, we only calculate the CrossEntropy loss. We evaluate the performance of our proposed model by adopting two different evaluation metric combinations for the two datasets. For IEMOCAP, we use WA (weighted accuracy) and UA (unweighted accuracy) as evaluation metrics. For MELD, we use Accuracy and Weighted average F1 as evaluation metrics. WA is the accuracy for all samples and UA is the accuracy for each emotion category. The evaluation metric combinations we selected are among the most used on the IEMOCAP dataset. The calculation of these four evaluation metrics are shown as follows.


(14) $$UA=\frac{\sum_{1}^{k}\frac{n_{i}}{N_{i}}}{k},\;WA=\frac{\sum_{1}^{k}n_{i}}{\sum_{1}^{k}N_{i}}$$

(15) $$\begin{array}{l} \text{Accuracy }=WA=\frac{\sum_{1}^{k}n_{i}}{\sum_{1}^{k}N_{i}}\\ \text{precision }=\frac{n_{i}}{M_{i}}\text{, recall }=\frac{n_{i}}{N_{i}},F1=\frac{2\times \text{ precision }\times \text{ recall }}{\text{ precision }+\text{ recall }}\\ \text{Weighted average }F1=\sum_{1}^{k}\frac{N_{i}\times F1_{i}}{N} \end{array}$$

where the *N*_*i*_ means the number of utterances in *i*_th_ class, the *M*_*i*_ represents the number of all emotions identified as *i*_*th*_ class, the *n*_*i*_ means the number of correctly recognized utterances in *i*_*th*_ class and *k* means the number of classes.

### 4.3. Ablation studies

In this study, we have conducted several ablation studies on the IEMOCAP and MELD datasets, respectively. The WA and UA evaluation metrics are used to evaluate our model on the IEMOCAP dataset. In addition, we evaluate our model by Accuracy and Weighted average F1 evaluation metrics on the MELD dataset.

#### 4.3.1. Results on the IEMOCAP dataset

To verify the impact of each mode, we train our proposed network using only audio features or text features as input without applying fusion modality. The audio features are wav2vec 2.0 features extracted by the pre-trained model wav2vec2-base. The text features are extracted by a pre-trained model roberta-base. Since the size of both audio features and text features are 768, the cross-modal attention mechanism of ResCMFA can effectively fuse the two features. From Table 3, we can see that the fusion of two features combines the advantages of both features and significantly improves the emotion recognition rate compared to a single feature. The addition of text features improved 3.35% in WA and 3.04% in UA compared to using only audio features.

**Table 3:** Comparison of results in different modalities on the IEMOCAP dataset.

**Models**	**WA**	**UA**
Only Roberta-base (baseline)	69.89%	69.27%
Only Wav2vec 2.0 (baseline)	78.66%	79.76%
Roberta-base + Wav2vec 2.0	82.01%	82.80%

In addition, we investigate the impact of the global-aware block for our proposed model. The addition of the global-aware block enables our proposed GCF^2^-Net to capture emotion information from fusion features at multiple scales. According to Table 4, we can see that adding global-aware blocks improves 1.09 and 1.07% in WA and UA, respectively. Thus, it can demonstrate that the global-aware block is able to extract more important emotional information to improve the performance of our model.

**Table 4:** Comparison of results in the global-aware block.

**Models**	**WA**	**UA**
W/O global-aware block	80.92%	81.73%
GCF^2^-Net (ours)	82.01%	82.80%

With the addition of global-aware, we also set up ablation experiments for the residual cross-modal fusion attention module (ResCMFA). Cross-modal attention mechanisms are multi-head attention mechanisms using different modal features as input. It can effectively fuse the features of two different modalities. Since we have two types of ResCMFA blocks that are placed in parallel. This allows to balance the weight of each modality feature. Therefore, we verify the effect of different numbers of the ResCMFA block layer in our proposed model. Table 5 shows the optimal model performance when having four layers of ResCMFA blocks (*m* = 4). However, the accuracy of the model decreases when *m* = 5. We consider *m* = 4 as our best choice.

**Table 5:** Comparison of results in different number of ResCMFA modules.

***m* = *i***	**WA**	**UA**
1	79.29%	79.92%
2	79.56%	80.79%
3	81.10%	82.16%
4	**82.01%**	**82.80%**
5	80.47%	80.95%

Bold value indicates comparison of results of ResCMFA blocks.

In addition, after determining the optimal model structure, we explore whether ASR as a secondary task contributes to emotion recognition. It is known that the hyperparameter α can control the intensity of CTC loss. Thus, we try to change α from 0 to 1 to obtain a different acceleration. Table 6 shows the effect of different values of α for our optimal model. We can learn that when α = 0.1, the positive impact of CTC loss is the largest. However, when α = 1, the addition of auxiliary tasks lowers the model recognition rate.

**Table 6:** Comparison of results in different values of α on the IEMOCAP dataset.

**α**	**WA**	**UA**
0	81.10%	81.56%
0.001	81.65%	81.88%
0.01	80.47%	81.19%
0.1	**82.01%**	**82.80%**
1	76.22%	77.05%

Bold value indicates comparison of results on the IEMOCAP dataset.

#### 4.3.2. Results on the MELD dataset

For MELD dataset, we also perform ablation experiments for audio features (wav2vec 2.0), text features (roberta-base), and cross-modal fusion features (wav2vec 2.0 features and text features). Table 7 shows the results of our proposed model on the MELD dataset using different modalities features. From this table, we can see that, unlike the IEMOCAP dataset, our model achieves a higher recognition rate for text features than audio features on the MELD dataset. The fusion of the two modal features improved the accuracy by 6.25% and the weighted average F1 by 6.63% compared to using only audio features.

**Table 7:** Comparison of results in different modalities on the MELD dataset.

**Models**	**Accuracy**	**Weighted average F1**
Only Roberta-base (baseline)	65.25%	62.82%
Only wav2vec 2.0 (baseline)	63.50%	60.85%
Roberta-base + wav2vec 2.0	69.75%	67.48%

Then, we perform ablation experiments for the global-aware blocks on the MELD dataset. Table 8 shows the experimental results for our model with or without the global-aware block. We can see that on the MELD dataset, the addition of the global-aware block improves the accuracy by 1.63% and the weighted average F1 by 1.75%. This further demonstrates the validity of the addition of the global-aware block to our proposed model.

**Table 8:** Comparison of results in the global-aware block on the MELD dataset.

**Models**	**Accuracy**	**Weighted average F1**
W/O global-aware block	68.12%	65.73%
GCF^2^-Net	69.75%	67.48%

Third, we conduct ablation experiments on ResCMFA block layers. With the ResCMFA block, we can effectively fuse the extracted audio features and text features. Table 9 shows the experimental results for our proposed model adopting different ResCMFA block layers on the MELD dataset. Unlike on the IEMOCAP dataset, our model achieves optimal results on the MELD dataset using two ResCMFA block layers (*m* = 2). Due to the large number of neutral categories on the MELD dataset. We speculate that too many ResCMFA block layers cause our proposed model to easily confuse neutral and other emotions.

**Table 9:** Comparison of result in different number of ResCMFA modules on MELD dataset.

***m* = *i***	**Accuracy**	**Weighted average F1**
1	68.32%	66.43%
2	**69.75%**	**67.48%**
3	67.83%	65.86%
4	68.58%	66.48%
5	66.83%	64.94%

Bold value indicates comparison of results of ResCMFA blocks.

Finally, we set up ablation experiments for the hyperparameter α to verify the effect of CTC loss on our proposed model. From Table 10, we can see that the optimal result is obtained when α is 0.1. The addition of the auxiliary task improved the accuracy by 1.67% and the weighted average F1 by 1.53% compared to without using ASR as an auxiliary task to calculate CTC loss. However, a large value of α will make our proposed model too dependent on CTC loss when updating parameters (only the training part).

**Table 10:** Comparison of results in different values of α on the MELD dataset.

**α**	**Accuracy**	**Weighted average F1**
0	68.08%	65.95%
0.001	68.24%	66.44%
0.01	68.73%	66.92%
0.1	**69.75%**	**67.48%**
1	63.77%	62.41%

Bold value indicates comparison of results on the MELD dataset.

### 4.4. Error analysis

**Figure 3:**
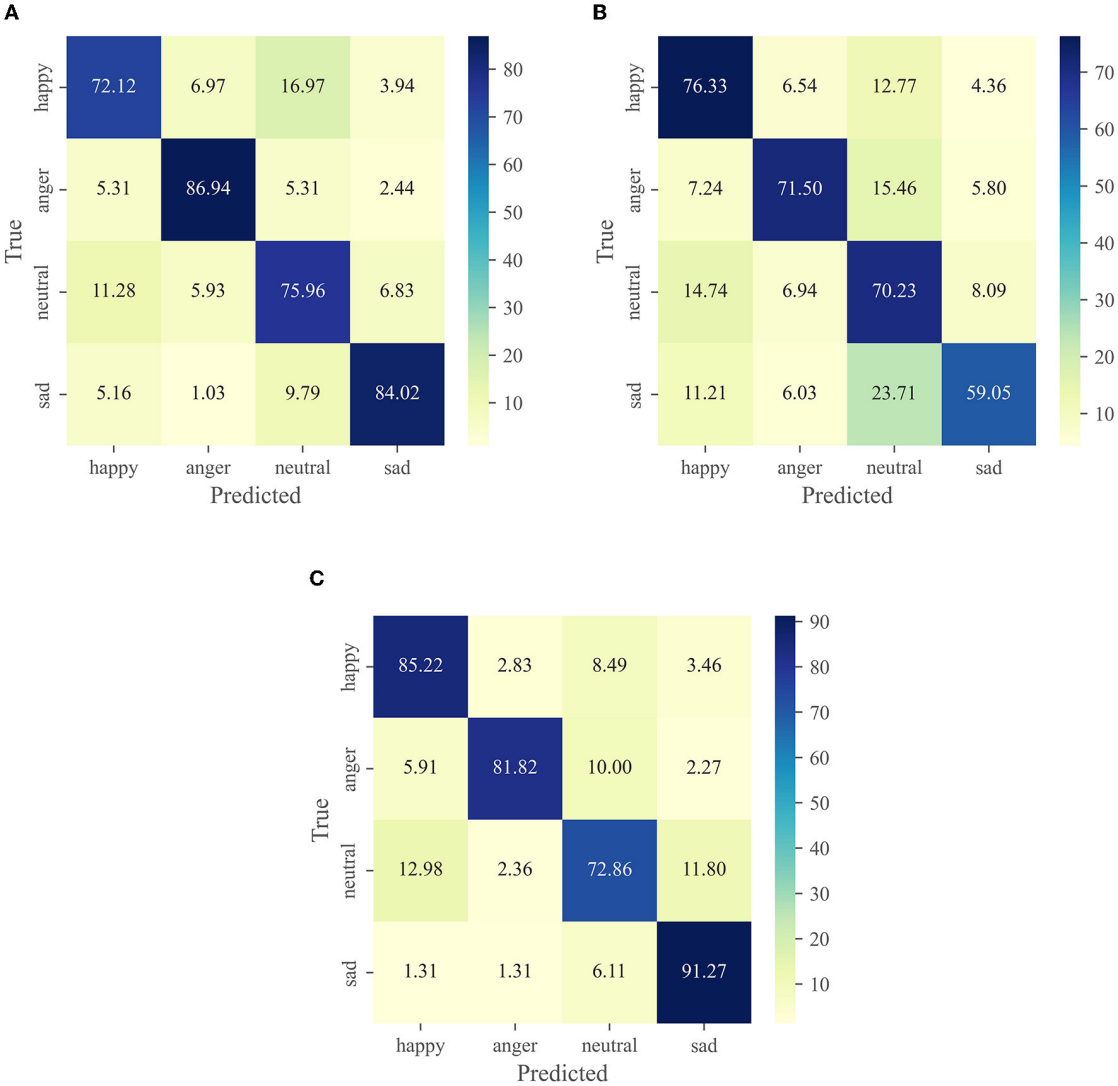
Experimental results in different modalities on the IEMOCAP dataset. **(A)** wav2vec 2.0. **(B)** Roberta-base. **(C)** Cross-modal function.

**Figure 4:**
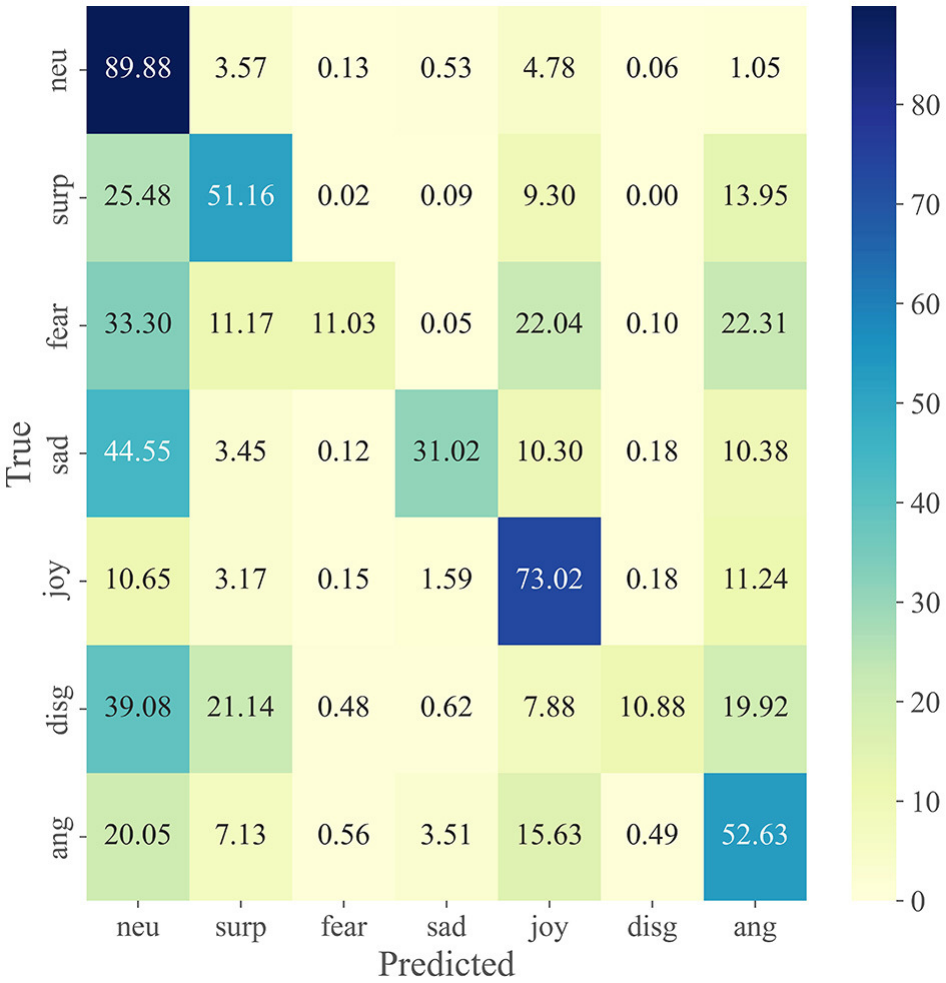
Experimental results in different modalities on the MELD dataset.

We visualize the performance and span of different modalities in different emotional categories through a confusion matrix. Figure 3 shows the confusion matrix for each modality on the IEMOCAP dataset, including (Wav2vec 2.0, Roberta-base and cross-modal fusion). Figure 4 shows the confusion matrix on the MELD dataset.

As can be seen in Figure 3A, it incorrectly confuses happy and neutral. Therefore, the recognition rate on these two emotions is far lower than the other two emotions, especially the recognition rate of anger reaches 86.94%. In general, most emotions are susceptible to confusion with neutral. Our observations agree with other studies reported on the IEMOCAP dataset (Yoon et al., 2018; Padi et al., 2022), which argued that neutral is located at the center of the activation space, making its discrimination from other categories more challenging. Compared with Figures 3A, B has excellent effectiveness in predicting happiness. This result is reasonable, compared to the audio signal data, happiness and other emotions have a more significant difference in word distribution and can provide more emotional information. On the other hand, sad has the worst prediction and it has 23.71% confusion with neutral.

The model in Figure 3C compensates for the shortcomings of the first two models (Figures 3A, B) by using the fusion of two modal features. We can see that the prediction rate for each emotion reaches 80% except for neutral. In particular, the prediction of sad reached 91.27%. Unfortunately, the recognition rate of anger and neutral is slightly reduced.

From Figure 4 we can see that the recognition rate of various emotions is extremely unbalanced. Neutral emotions had the highest recognition rate of 89.94%, however, the recognition rates for fear and disgust were only 11.03 and 10.88%, respectively. This may be because the number of neutral in the test set reached almost 50%, resulting in many emotion categories being identified as neutral. And the number of fear and disgust emotions is too sparse leading to extremely few other emotions identified as fear and disgust. The same conclusions are presented in Zhang et al. (2019); Kim and Vossen (2021); Song et al. (2022).

### 4.5. Comparative analysis

As shown in Table 11, we compare cross-modal emotion recognition models in WA and UA using the same modality data. The IEMOCAP dataset is not equally distributed, so the WA and UA enable to evaluate the classification ability of the model. It is worth mentioning that we choose the same audio and text features as Srivastava et al. (2022). However, the addition of the ResCMFA module and the global-aware block allows our proposed GCF^2^-Net model to improve by 4.37% in WA. This further demonstrates that the ResCMFA module and global-aware can better fuse the emotional information of different modalities. In addition, our proposed GCF^2^-Net model improves WA by 1.65% and UA by 1.10% over previous state-of-the-art methods (Li et al., 2022). It can see that our model achieves state-of-the-art experimental results on the WA and UA. The comparison further demonstrates the validity of our proposed model. Compared with existing cross-modal approaches, our proposed GCF^2^-Net network on MELD dataset achieve a significant improvement over the state-of-the-art cross-modal emotion recognition. Table 12 shows that our proposed model improves the accuracy by 1.90% and the weighted average F1 by 0.77% compared to the previous optimal model (Chudasama et al., 2022). In addition, we discuss the calculation complexity of our proposed model. The average duration of audio on the IEMOCAP and MELD datasets is 4.5 seconds. Therefore, we choose the audio of 4.5 s and the corresponding text as input to calculate the model complexity. We set the audio size (245,768) and the text size (22,768), our proposed model produces 128.63 MB parameters, the input size is 0.78 MB, and the forward/backward process size is 83.94 MB. Our further work will also consider how to reduce the model parameters.

**Table 11:** Comparison of experiment results of different methods on the IEMOCAP dataset.

**Method**	**WA**	**UA**
Xu et al. (2019)	70.40%	69.50%
Liu et al. (2020)	72.40%	70.10%
Makiuchi et al. (2021)	73.50%	73.00%
Cai et al. (2021)	78.15%	–
Morais et al. (2022)	77.36%	77.76%
Srivastava et al. (2022)	77.64%	–
Qian and Han (2022)	76.06%	77.45%
Li et al. (2022)	80.36%	81.70%
GCF^2^-Net	**82.01%**	**82.80%**

Bold value indicates comparison of results of different methods on the IEMOCAP dataset.

**Table 12:** Comparison of experiment results of different methods on the MELD dataset.

**Method**	**Accuracy**	**Weighted average F1**
Zhang et al. (2019)	–	57.40%
Mao et al. (2020)	65.66%	63.55%
Siriwardhana et al. (2020)	64.30%	63.90%
Lian et al. (2021)	62.00%	60.50%
Xie et al. (2021)	65.00%	64.00%
Hu et al. (2022)	65.09%	65.51%
Song et al. (2022)	–	66.50%
Chudasama et al. (2022)	67.85%	66.71%
GCF^2^-Net	**69.75%**	**67.48%**

Bold value indicates comparison of results of different methods on the MELD dataset.

In summary, compared with other existing methods, our proposed model greatly improves all metrics. This can be attributed to the fact that the wav2vec 2.0 features we extracted do not require trimming the audio, thus preserving a large amount of emotional information. According to Li et al. (2022), audio is clipped into 5 s and texts are clipped into 512 tokens by trimming or padding. Second, the addition of global-aware enables our proposed model to extract more global information. Finally, the CTC loss function is used to back-propagate the gradient and further improves the accuracy.

### 4.6. Discussion

Our proposed GCF^2^-Net model achieves state-of-the-art results on both the IEMOCAP and MELD datasets. In our study, we chose to integrate textual information into audio messages because audio messages and textual information are closely related to our communication. Tables 3, 7 show that cross-modal emotion recognition achieves superior results than speech emotion recognition or text emotion recognition. Figure 3 confusion matrix further shows the superiority of cross-modal emotion recognition on the IEMOCAP dataset. Speech emotion recognition performs well on anger and sad, but poorly on happy and neutral. It is easy to confuse neutral and happy. In contrast, text emotion recognition performs well on happy and neutral. In Figure 3C, we can observe that the best performance is achieved for cross-modal sentiment recognition compared to using only audio modality features. It greatly improves the rec ognition rate of happy and sad, which reflects the synergistic property between audio and text.

As can be seen from the ablation study in Section 4.3, each block in our model plays a crucial role in the experimental results. Tables 4, 8 show that cross-modal emotion recognition achieves superior results than speech emotion recognition or text motion recognition. First, we designed a ResCMFA block based on CMA to fuse audio features (wav2vec 2.0) and text features (roberta-base) from transfer learning. From Tables 5, 9 we can see that our proposed model achieves optimal results in the 4-layer (IEMOCAP dataset) and 2-layer (MELD dataset) ResCMFA block layers, respectively. In addition, according to the Tables 4, 8 we can see that the inclusion of the global-aware block achieves superior results on both datasets. The global-aware block is able to capture emotional information in cross-modal fusion features from different scales. Finally, the parameters of the model are updated in the training part by computing CTC loss. From Tables 6, 10 we can see that different intensity of CTC loss has different effects on our proposed GCF^2^-Net model.

In addition, we discuss the calculation complexity of our proposed model. The average duration of audio on the IEMOCAP and MELD datasets is 4.5 s. Therefore, we choose the audio of 4.5 s and the corresponding text as input to calculate the model complexity. We set the audio size (245,768) and the text size (22,768), our proposed model produces 128.63 MB parameters, the input size is 0.78 MB, and the forward/backward process size is 83.94 MB. Our further work will also consider how to reduce the model parameters.

## 5. Conclusion

In this paper, we propose a novel deep learning architecture to recognize speech emotion called global-aware cross-modal feature Fusion Network (GCF^2^-Net). Firstly, we construct a residual cross-modal fusion attention (ResCMFA) module that helps the network to extract rich features from audio and text. Then, the global-aware block is added after ResCMFA module to extract emotion-rich features further globally. We also introduce ASR as an auxiliary task to calculate CTC loss. Finally, experimental results on the IEMOCAP dataset demonstrate that the ResCMFA module, global-aware block, ASR to calculate CTC loss all improve the performance of the model. In future work, we will explore more methods to improve the performance of cross-modal emotion recognition. We endeavor to improve people's happiness by applying our approach to all areas of society, such as healthcare, autonomous driving, smart Q&A, etc.

## Data availability statement

Publicly available datasets were analyzed in this study. This data can be found here: https://sail.usc.edu/iemocap/.

## Author contributions

FL and JL designed the experiments and prepared the manuscript. LW assisted in project supervision and carried out the experiments. WL and XS analyzed the results. All authors contributed to the article and approved the submitted version.

## References

[B1] Ahn C.-S. Kasun C. Sivadas S. Rajapakse J. (2022). “Recurrent multi-head attention fusion network for combining audio and text for speech emotion recognition,” in Proc. Interspeech 2022 (Incheon), 744–748.

[B2] Babu P. A. Nagaraju V. S. Vallabhuni R. R. (2021). “Speech emotion recognition system with librosa,” in 2021 10th IEEE International Conference on Communication Systems and Network Technologies (CSNT) (Bhopal: IEEE), 421–424.

[B3] Baevski A. Zhou Y. Mohamed A. Auli M. (2020). wav2vec 2.0: a framework for self-supervised learning of speech representations. Adv. Neural Inf. Process. Syst. 33, 12449–12460. 10.5555/3495724.3496768

[B4] Cai X. Yuan J. Zheng R. Huang L. Church K. (2021). “Speech emotion recognition with multi-task learning,” in Interspeech (Brno).

[B5] Cambria E. Hazarika D. Poria S. Hussain A. Subramanyam R. (2017). “Benchmarking multimodal sentiment analysis,” in International conference on computational linguistics and intelligent text processing (Budapest: Springer), 166–179.

[B6] Cao Q. Hou M. Chen B. Zhang Z. Lu G. (2021). “Hierarchical network based on the fusion of static and dynamic features for speech emotion recognition,” in ICASSP 2021-2021 IEEE International Conference on Acoustics, Speech and Signal Processing (ICASSP) (Toronto, ON: IEEE), 6334–6338.

[B7] Chakravarthi B. Ng S.-C. Ezilarasan M. Leung M.-F. (2022). Eeg-based emotion recognition using hybrid cnn and lstm classification. Front. Comp. Neurosci. 16, 1019776. 10.3389/fncom.2022.1019776 36277613 PMC9585893

[B8] Chan W. Jaitly N. Le Q. Vinyals O. (2016). “Listen, attend and spell: a neural network for large vocabulary conversational speech recognition,” in 2016 IEEE international conference on acoustics, speech and signal processing (ICASSP) (Shanghai: IEEE), 4960–4964.

[B9] Chang H. Zong Y. Zheng W. Tang C. Zhu J. Li X. (2022). Depression assessment method: an eeg emotion recognition framework based on spatiotemporal neural network. Front. Psychiatry 12, 2620. 10.3389/fpsyt.2021.837149 35368726 PMC8967371

[B10] Chang H. Zong Y. Zheng W. Xiao Y. Wang X. Zhu J.. (2023). Eeg-based major depressive disorder recognition by selecting discriminative features via stochastic search. J. Neural Eng. 20, 026021. 10.1088/1741-2552/acbe20 36812637

[B11] Chen Y. Yuan J. You Q. Luo J. (2018). “Twitter sentiment analysis via bi-sense emoji embedding and attention-based lstm,” in Proceedings of the 26th ACM international conference on Multimedia (New York, NY), 117–125. 10.1145/3240508.3240533

[B12] Chen Z. Chen S. Wu Y. Qian Y. Wang C. Liu S.. (2022). “Large-scale self-supervised speech representation learning for automatic speaker verification,” in ICASSP 2022-2022 IEEE International Conference on Acoustics, Speech and Signal Processing (ICASSP) (Singapore: IEEE), 6147–6151.

[B13] Cho J. Pappagari R. Kulkarni P. Villalba J. Carmiel Y. Dehak N. (2019). Deep neural networks for emotion recognition combining audio and transcripts. arXiv. 247–251. 10.21437/Interspeech.2018-2466

[B14] Choi W. Y. Song K. Y. Lee C. W. (2018). “Convolutional attention networks for multimodal emotion recognition from speech and text data,” in Proceedings of grand challenge and workshop on human multimodal language (Challenge-HML) (Melbourne, VI), 28–34.

[B15] Chudasama V. Kar P. Gudmalwar A. Shah N. Wasnik P. Onoe N. (2022). “M2fnet: Multi-modal fusion network for emotion recognition in conversation,” in Proceedings of the IEEE/CVF Conference on Computer Vision and Pattern Recognition (Vancouver, BC), 4652–4661.

[B16] Devlin J. Chang M.-W. Lee K. Toutanova K. (2018). Bert: Pre-training of deep bidirectional transformers for language understanding. arXiv. 4171–4186. 10.18653/v1/N19-1423

[B17] Dissanayake V. Tang V. Elvitigala D. S. Wen E. Wu M. Nanayakkara S. (2022). Troi: Towards understanding users perspectives to mobile automatic emotion recognition system in their natural setting. Proc. ACM Hum. Comp. Interact. 6, 1–22. 10.1145/3546738

[B18] Fan W. Xu X. Cai B. Xing X. (2022). Isnet: Individual standardization network for speech emotion recognition. IEEE/ACM Transact. Audio Speech Lang. Process. 1803–1814. 10.1109/TASLP.2022.3171965

[B19] Gao M. Dong J. Zhou D. Zhang Q. Yang D. (2019). “End-to-end speech emotion recognition based on one-dimensional convolutional neural network,” in Proceedings of the 2019 3rd International Conference on Innovation in Artificial Intelligence (Suzhou), 78–82.

[B20] Georgiou E. Papaioannou C. Potamianos A. (2019). “Deep hierarchical fusion with application in sentiment analysis,” in INTERSPEECH (Graz), 1646–1650. 10.21437/Interspeech.2019-3243

[B21] Girish K. V. Konjeti S. Vepa J. (2022). “Interpretabilty of speech emotion recognition modelled using self-supervised speech and text pre-trained embeddings,” in Interspeech (Incheon), 4496–4500.

[B22] Han K. Yu D. Tashev I. (2014). “Speech emotion recognition using deep neural network and extreme learning machine,” in Interspeech (Singapore).

[B23] Han Z. Chang H. Zhou X. Wang J. Wang L. Shao Y. (2022). E2ennet: an end-to-end neural network for emotional brain-computer interface. Front. Comp. Neurosci. 16, 942979. 10.3389/fncom.2022.942979 36034935 PMC9413837

[B24] Hartmann K. Siegert I. Philippou-Hübner D. Wendemuth A. (2013). Emotion detection in hci: from speech features to emotion space. IFAC Proceedings Volumes 46, 288–295.

[B25] Hu G. Lin T.-E. Zhao Y. Lu G. Wu Y. Li Y. (2022). Unimse: Towards unified multimodal sentiment analysis and emotion recognition. arXiv. 7837–7851. 10.48550/arXiv.2211.11256

[B26] Irsoy O. Cardie C. (2014). “Opinion mining with deep recurrent neural networks,” in Proceedings of the 2014 conference on empirical methods in natural language processing (EMNLP) (Doha), 720–728.

[B27] Jain M. Narayan S. Balaji P. Bhowmick A. Muthu R. K. (2020). Speech emotion recognition using support vector machine. arXiv. 1–6. 10.48550/arXiv.2002.07590

[B28] Kandali A. B. Routray A. Basu T. K. (2008). “Emotion recognition from assamese speeches using mfcc features and gmm classifier,” in TENCON 2008-2008 IEEE Region 10 Conference (Hyderabad: IEEE), 1–5.

[B29] Khaireddin Y. Chen Z. (2021). Facial emotion recognition: State of the art performance on fer2013. arXiv. 1–9. 10.48550/arXiv.2105.03588

[B30] Kim T. Vossen P. (2021). Emoberta: Speaker-aware emotion recognition in conversation with roberta. arXiv. 1–4. 10.48550/arXiv.2108.12009

[B31] Kishore K. K. Satish P. K. (2013). “Emotion recognition in speech using mfcc and wavelet features,” in 2013 3rd IEEE International Advance Computing Conference (IACC) (IEEE), 842–847. 10.1109/IAdCC.2013.6514336

[B32] Ko B. C. (2018). A brief review of facial emotion recognition based on visual information. Sensors 18, 401. 10.3390/s18020401 29385749 PMC5856145

[B33] Krishna D. Patil A. (2020). “Multimodal emotion recognition using cross-modal attention and 1d convolutional neural networks,” in Interspeech (Shanghai), 4243–4247.

[B34] Kwon O.-W. Chan K. Hao J. Lee T.-W. (2003). “Emotion recognition by speech signals,” in Eighth European Conference on Speech Communication and Technology (Geneva).

[B35] Li J. Ji D. Li F. Zhang M. Liu Y. (2020). “Hitrans: a transformer-based context-and speaker-sensitive model for emotion detection in conversations,” in Proceedings of the 28th International Conference on Computational Linguistics (Barcelona), 4190–4200.

[B36] Li J. Wang S. Chao Y. Liu X. Meng H. (2022). Context-aware multimodal fusion for emotion recognition. Proc. Interspeech 2022 (Incheon), 2013–2017. 10.21437/Interspeech.2022-1059233801739

[B37] Lian Z. Liu B. Tao J. (2021). Ctnet: conversational transformer network for emotion recognition. IEEE/ACM Transact. Audio Speech Lang. Proc. 29, 985–1000. 10.1109/TASLP.2021.3049898

[B38] Liu H. Dai Z. So D. Le Q. V. (2021). Pay attention to mlps. Adv. Neural Inf. Process. Syst. 34, 9204–9215. 10.48550/arXiv.2105.08050

[B39] Liu P. Li K. Meng H. (2020). “Group gated fusion on attention-based bidirectional alignment for multimodal emotion recognition,” in INTERSPEECH, 379–383.

[B40] Liu Y. Ott M. Goyal N. Du J. Joshi M. Chen D.. (2019). Roberta: A robustly optimized bert pretraining approach. arXiv. 1–13. 10.48550/arXiv.1907.11692

[B41] Makiuchi M. R. Uto K. Shinoda K. (2021). “Multimodal emotion recognition with high-level speech and text features,” in 2021 IEEE Automatic Speech Recognition and Understanding Workshop (ASRU) (Cartagena: IEEE), 350–357.

[B42] Mao Y. Sun Q. Liu G. Wang X. Gao W. Li X.. (2020). Dialoguetrm: Exploring the intra-and inter-modal emotional behaviors in the conversation. arXiv. 2694–2704. 10.18653/v1/2021.findings-emnlp.22936568019

[B43] Mikolov T. Chen K. Corrado G. Dean J. (2013). Efficient estimation of word representations in vector space. arXiv. 1–12. 10.48550/arXiv.1301.378131752376

[B44] Morais E. Hoory R. Zhu W. Gat I. Damasceno M. Aronowitz H. (2022). “Speech emotion recognition using self-supervised features,” in ICASSP 2022-2022 IEEE International Conference on Acoustics, Speech and Signal Processing (ICASSP) (Singapore: IEEE), 6922–6926. 10.1109/ICASSP43922.2022.9747870

[B45] Nwe T. L. Foo S. W. De Silva L. C. (2003). Speech emotion recognition using hidden markov models. Speech Commun. 41, 603–623. 10.1016/S0167-6393(03)00099-2

[B46] Padi S. Sadjadi S. O. Manocha D. Sriram R. D. (2022). Multimodal emotion recognition using transfer learning from speaker recognition and bert-based models. arXiv. 407–414. 10.21437/Odyssey.2022-57

[B47] Pang B. Lee L. (2004). A sentimental education: Sentiment analysis using subjectivity summarization based on minimum cuts. arXiv. 1–8. 10.3115/1218955.1218990

[B48] Pennington J. Socher R. Manning C. D. (2014). “Glove: Global vectors for word representation,” in Proceedings of the 2014 Conference on Empirical Methods in Natural Language Processing (EMNLP) (Doha), 1532–1543.

[B49] Pepino L. Riera P. Ferrer L. (2021). Emotion recognition from speech using wav2vec 2.0 embeddings. arXiv. 3400–3404. 10.21437/Interspeech.2021-703

[B50] Pepino L. Riera P. Ferrer L. Gravano A. (2020). “Fusion approaches for emotion recognition from speech using acoustic and text-based features,” in ICASSP 2020-2020 IEEE International Conference on Acoustics, Speech and Signal Processing (ICASSP) (Graz: IEEE), 6484–6488.

[B51] Poria S. Cambria E. Hazarika D. Majumder N. Zadeh A. Morency L.-P. (2017). “Context-dependent sentiment analysis in user-generated videos,” in Proceedings of the 55th Annual Meeting of the Association for Computational Linguistics (volume 1: Long papers) (Barcelona), 873–883.

[B52] Poria S. Hazarika D. Majumder N. Naik G. Cambria E. Mihalcea R. (2018a). Meld: a multimodal multi-party dataset for emotion recognition in conversations. arXiv. 527–536. 10.18653/v1/P19-1050

[B53] Poria S. Majumder N. Hazarika D. Cambria E. Gelbukh A. Hussain A. (2018b). Multimodal sentiment analysis: addressing key issues and setting up the baselines. IEEE Intell. Syst. 33, 17–25. 10.1109/MIS.2018.2882362

[B54] Qian F. Han J. (2022). Contrastive regularization for multimodal emotion recognition using audio and text. arXiv. 1–5. 10.48550/arXiv.2211.10885

[B55] Ramet G. Garner P. N. Baeriswyl M. Lazaridis A. (2018). “Context-aware attention mechanism for speech emotion recognition,” in 2018 IEEE Spoken Language Technology Workshop (SLT) (Brno: IEEE), 126–131.

[B56] Schuller B. W. (2018). Speech emotion recognition: two decades in a nutshell, benchmarks, and ongoing trends. Commun. ACM 61, 90–99. 10.1145/3129340

[B57] Sebastian J. Pierucci P.. (2019). “Fusion techniques for utterance-level emotion recognition combining speech and transcripts,” in Interspeech (Graz), 51–55.

[B58] Sheng D. Wang D. Shen Y. Zheng H. Liu H. (2020). “Summarize before aggregate: a global-to-local heterogeneous graph inference network for conversational emotion recognition,” in Proceedings of the 28th International Conference on Computational Linguistics (Barcelona), 4153–4163.

[B59] Siriwardhana S. Kaluarachchi T. Billinghurst M. Nanayakkara S. (2020). Multimodal emotion recognition with transformer-based self supervised feature fusion. IEEE Access 8, 176274–176285. 10.1109/ACCESS.2020.3026823

[B60] Song X. Zang L. Zhang R. Hu S. Huang L. (2022). “Emotionflow: capture the dialogue level emotion transitions,” in ICASSP 2022-2022 IEEE International Conference on Acoustics, Speech and Signal Processing (ICASSP) (IEEE), 8542–8546.

[B61] Sreeshakthy M. Preethi J. (2016). Classification of human emotion from deap eeg signal using hybrid improved neural networks with cuckoo search. BRAIN. Broad Res. Artif. Intell. Neurosci. 6, 60–73.

[B62] Srivastava H. Ghosh S. Umesh S. (2022). Mmer: Multimodal multi-task learning for emotion recognition in spoken utterances. arXiv.

[B63] Sun L. Liu B. Tao J. Lian Z. (2021). “Multimodal cross-and self-attention network for speech emotion recognition,” in ICASSP 2021-2021 IEEE International Conference on Acoustics, Speech and Signal Processing (ICASSP) (IEEE), 4275–4279.

[B64] Tripathi S. Tripathi S. Beigi H. (2018). Multi-modal emotion recognition on iemocap dataset using deep learning. arXiv. 1–5. 10.48550/arXiv.1804.05788

[B65] Venkateswarlu S. Kumar N. U. Veeraswamy D. Vijay V. (2022). “Speech intelligibility quality in telugu speech patterns using a wavelet-based hybrid threshold transform method,” in Intelligent Systems and Sustainable Computing (Hyderabad: Springer), 449–462.

[B66] Ververidis D. Kotropoulos C. (2006). Emotional speech recognition: resources, features, and methods. Speech commun. 48, 1162–1181. 10.1016/j.specom.2006.04.003

[B67] Wang Y. Boumadane A. Heba A. (2021). A fine-tuned wav2vec 2.0/hubert benchmark for speech emotion recognition, speaker verification and spoken language understanding. arXiv. 1–7. 10.48550/arXiv.2111.02735

[B68] Wang Y. Shen Y. Liu Z. Liang P. P. Zadeh A. Morency L.-P. (2019). “Words can shift: dynamically adjusting word representations using nonverbal behaviors,” in Proceedings of the AAAI Conference on Artificial Intelligence. Vol. 33 (Honolulu, HI), 7216–7223. 32219010 PMC7098710

[B69] Wang Y. Zhang J. Ma J. Wang S. Xiao J. (2020). “Contextualized emotion recognition in conversation as sequence tagging,” in Proceedings of the 21th Annual Meeting of The Special Interest Group on Discourse and Dialogue, 186–195.

[B70] Wu M. Teng W. Fan C. Pei S. Li P. Lv Z. (2023). An investigation of olfactory-enhanced video on eeg-based emotion recognition. IEEE Transact. Neural Syst. Rehabil. Eng. 31, 1602–1613. 10.1109/TNSRE.2023.3253866 37028354

[B71] Wu W. Zhang C. Woodland P. C. (2021). “Emotion recognition by fusing time synchronous and time asynchronous representations,” in ICASSP 2021-2021 IEEE International Conference on Acoustics, Speech and Signal Processing (ICASSP) (Toronto, ON: IEEE), 6269–6273.

[B72] Xie B. Sidulova M. Park C. H. (2021). Robust multimodal emotion recognition from conversation with transformer-based crossmodality fusion. Sensors 21, 4913. 10.3390/s21144913 34300651 PMC8309929

[B73] Xu H. Zhang H. Han K. Wang Y. Peng Y. Li X. (2019). Learning alignment for multimodal emotion recognition from speech. arXiv. 3569–3573. 10.21437/Interspeech.2019-3247

[B74] Xu M. Zhang F. Cui X. Zhang W. (2021). “Speech emotion recognition with multiscale area attention and data augmentation,” in ICASSP 2021-2021 IEEE International Conference on Acoustics, Speech and Signal Processing (ICASSP) (Toronto, ON: IEEE), 6319–6323.

[B75] Yang S.-W. Chi P.-H. Chuang Y.-S. Lai C.-I. J. Lakhotia K. Lin Y. Y.. (2021). Superb: speech processing universal performance benchmark. arXiv. 1194–1198. 10.21437/Interspeech.2021-1775

[B76] Yang X. Che H. Leung M.-F. Liu C. (2022). Adaptive graph nonnegative matrix factorization with the self-paced regularization. Appl. Intell. 1–18. 10.1007/s10489-022-04339-w

[B77] Yoon S. Byun S. Dey S. Jung K. (2019). “Speech emotion recognition using multi-hop attention mechanism,” in ICASSP 2019-2019 IEEE International Conference on Acoustics, Speech and Signal Processing (ICASSP) (Brighton: IEEE), 2822–2826.

[B78] Yoon S. Byun S. Jung K. (2018). “Multimodal speech emotion recognition using audio and text,” *in 2018 IEEE Spoken Language Technology Workshop (SLT)* (IEEE), 112–118.

[B79] Zhang D. Wu L. Sun C. Li S. Zhu Q. Zhou G. (2019). “Modeling both context-and speaker-sensitive dependence for emotion detection in multi-speaker conversations,” in IJCAI (Macao), 5415–5421.

[B80] Zhang J. Li C. Kosov S. Grzegorzek M. Shirahama K. Jiang T.. (2021). Lcu-net: a novel low-cost u-net for environmental microorganism image segmentation. Patt. Recogn. 115, 107885. 10.1016/j.patcog.2021.107885

[B81] Zhang M. Chen Y. Lin Y. Ding H. Zhang Y. (2022). Multichannel perception of emotion in speech, voice, facial expression, and gesture in individuals with autism: a scoping review. J. Speech Lang. Hear. Res. 65, 1435–1449. 10.1044/2022_JSLHR-21-00438 35316079

[B82] Zhang X. Zheng X. (2016). “Comparison of text sentiment analysis based on machine learning,” in 2016 15th International Symposium on Parallel and Distributed Computing (ISPDC) (Fuzhou), 230–233.

[B83] Zhao Z. Wang Y. Wang Y. (2022). Multi-level fusion of wav2vec 2.0 and bert for multimodal emotion recognition. arXiv. 4725–4729. 10.21437/Interspeech.2022-10230

[B84] Zhu W. Li X. (2022). “Speech emotion recognition with global-aware fusion on multi-scale feature representation,” in ICASSP 2022-2022 IEEE International Conference on Acoustics, Speech and Signal Processing (ICASSP) (Singapore: IEEE), 6437–6441.

